# Associations of plasma lipids, lipoproteins, and cardiovascular outcomes with climatic variations in a large Brazilian population of Campinas, São Paulo state: an eight-year study

**DOI:** 10.1590/1414-431X2021e11035

**Published:** 2021-08-06

**Authors:** W. Corozolla, V.H.S. Zago, F.A.L. Marson, A.M.H. de Avila, P.D.P. Costa, L.S. Teixeira, F. Dalpino, E.C. de Faria

**Affiliations:** 1 Departamento de Patologia Clínica, Laboratório de Lípides, Núcleo de Medicina e Cirurgia Experimental, Faculdade de Ciências Médicas, Universidade Estadual de Campinas, Campinas, SP, Brasil; 2 Pontifícia Universidade Católica de Campinas, Campinas, SP, Brasil; 3 Laboratório de Biologia Celular e Molecular de Tumores e Compostos Bioativos, Universidade São Francisco, Bragança Paulista, SP, Brasil; 4 Laboratório de Genética Humana e Genética Médica, Universidade São Francisco, Bragança Paulista, SP, Brasil; 5 Centro de Pesquisas Meteorológicas e Climáticas Aplicadas è Agricultura, Universidade Estadual de Campinas, Campinas, SP, Brasil; 6 Departamento de Engenharia de Computação e Automação, Faculdade de Engenharia Elétrica e de Computação, Universidade Estadual de Campinas, Campinas, SP, Brasil

**Keywords:** Atherosclerotic cardiovascular disease, Climatic variations, Lipoproteins, Seasonality

## Abstract

In this eight-year retrospective study, we evaluated the associations between climatic variations and the biological rhythms in plasma lipids and lipoproteins in a large population of Campinas, São Paulo state, Brazil, as well as temporal changes of outcomes of cardiovascular hospitalizations. Climatic variables were obtained at the Center for Meteorological and Climatic Research Applied to Agriculture (University of Campinas - Unicamp, Brazil). The plasma lipid databases surveyed were from 27,543 individuals who had their lipid profiles assessed at the state university referral hospital in Campinas (Unicamp). The frequency of hospitalizations was obtained from the Brazilian Public Health database (DATASUS). Temporal statistical analyses were performed using the methods Cosinor or Friedman (ARIMA) and the temporal series were compared by cross-correlation functions. In normolipidemic cases (n=11,892), significantly different rhythmicity was observed in low-density lipoprotein (LDL)- and high-density lipoprotein (HDL)-cholesterol (C) both higher in winter and lower in summer. Dyslipidemia (n=15,651) increased the number and amplitude of lipid rhythms: LDL-C and HDL-C were higher in winter and lower in summer, and the opposite occurred with triglycerides. The number of hospitalizations showed maximum and minimum frequencies in winter and in summer, respectively. A coincident rhythmicity was observed of lower temperature and humidity rates with higher plasma LDL-C, and their temporal series were inversely cross-correlated. This study shows for the first time that variations of temperature, humidity, and daylight length were strongly associated with LDL-C and HDL-C seasonality, but moderately to lowly associated with rhythmicity of atherosclerotic outcomes. It also indicates unfavorable cardiovascular-related changes during wintertime.

## Introduction

Coronary artery disease (CAD) is the main cause of morbimortality in Brazil (1). Since dyslipidemias are a strong risk factor for CAD, plasma lipid profiles, which are indispensable for the diagnosis and treatment of dyslipidemias (2), have been the object of much research (3). Moreover, the outcomes of cardiovascular diseases are also affected by the environment and climate (4,5). The evaluation of effects of seasonal variations on the biochemical parameters routinely analyzed in clinical laboratories is poor, although these rhythms could impact medical decisions (6). In addition, very few studies determined the seasonality of clinical manifestations of atherosclerosis (7,8). This led us to develop this large population study in Campinas, São Paulo state, Brazil, to quantify the biological rhythms in plasma lipids and lipoproteins in normolipidemic and dyslipidemic individuals and evaluate the associations with the variations of climatic parameters. We also investigated whether the frequency of atherosclerotic cardiovascular outcomes during the same period were associated with climatic variations.

Furthermore, we verified the possible associations between these rhythms by cross-correlation analyses of their respective temporal series, using chronobiological and temporal tools to improve understanding of the lipid rhythmic behavior.

## Material and Methods

### Study population and location

This was a retrospective study carried out with data from eight years, 1996 to 2003, of a large population sample in Brazil to evaluate the rhythmicity of their plasma lipids and lipoproteins. The population sample was of individuals registered at the state university referral hospital in Campinas (Hospital de Clínicas, Universidade Estadual de Campinas - Unicamp).

The population of Campinas is 1,080,113 inhabitants, with an average monthly salary of US$892.00, 78.6% are Caucasian and 20.7% are Afro-descendants (9). The outpatient study population, totaling 27,543 participants, included individuals of both sexes and all age groups, with a large racial miscegenation and a very heterogeneous socioeconomic background.

### Lipid and lipoprotein data

The 12-h fasting plasma laboratory exams were performed in an automated chemical analyzer under standardized conditions, following the manufacturer's instructions: Roche Diagnostics^®^ reagents (Mannheim, Germany). Control materials were systematically used to determine analytic variability and to maintain accuracy. Only one lipid profile per individual per year was chosen at random among all ambulatory participants to be analyzed.

The number of exams performed was 57,187 each for cholesterol (C), low-density lipoprotein-cholesterol (LDL-C), high-density lipoprotein-cholesterol (HDL-C), and triglycerides (TG), totaling 228,748 measurements. The ranges of results were: 26 to 1,304 mg/dL for cholesterol, 0 to 1,722 mg/dL for LDL-C, 0 to 272 mg/dL for HDL-C, and 4 to 3,880 mg/dL for TG.

Normolipidemic and dyslipidemic groups were classified according to the cutoff limits established by the National Cholesterol Education Program (NCEP) (10) and adopted by the Brazilian Guidelines for Dyslipidemia and Atherosclerosis prevention (11) for adults: cholesterol <200 mg/dL, LDL-C <130 mg/dL, HDL-C between 40 and 67 mg/dL, and TG <150 mg/dL. The cutoff limits adopted for children and adolescents were those recommended by Kwiterovich (12): cholesterol <170 mg/dL, LDL-C <110 mg/dL, HDL-C >45 mg/dL, and TG <75 mg/dL (below 10 years) and <90 mg/dL (between 10 and 19 years).

### Database of climatic variables

During the same eight-year period, data on climatic variables were collected, which were: minimum temperature, maximum temperature, and temperature amplitude (°C); minimum and maximum relative humidity (%); and daily luminosity, defined as light and dark cycles (h:min). These data were collected every 10 min, daily, by climate sensors from the Meteorological Station of the Center for Meteorological and Climate Research Applied to Agriculture (CEPAPRI), located at Unicamp at 22° 48' 56 "S, 47° 03' 28" W, at an elevation of 664 meters.

The meteorological definition of the seasons divided into three-month periods in Brazil is as follows: summer (December, January, February), autumn (March, April, May), winter (June, July, August), and spring (September, October, November) according to the Department of Astronomy of the Institute of Astronomy, Geophysics and Atmospheric Sciences (IAG/USP) (13).

### Outcomes of cardiovascular hospitalizations

Data from the same eight-year period about the frequency of hospitalizations from atherosclerotic cardiovascular outcomes defined by the International Classification of Diseases (ICD 9) in the city of Campinas was collected from the Information Technology Department of the Public Health Care System (DATASUS), to measure their rhythmicity. We selected as main cardiovascular outcomes atherosclerosis, stroke, myocardial infarction, and other vascular diseases. The outcomes comprised 23,434 cases of hospitalizations due to the following conditions: atherosclerosis (ICD 440) 3,261 cases, stroke (ICD 434) 11,145 cases, myocardial infarction (ICD 410) 6,343 cases, and other vascular diseases (ICD 443) 2,685 cases.

### Statistical analysis and data cleaning

Outlier detection analysis was performed by calculating the percentile values for each measurement, considering the total group of individuals. The outliers for each variable were defined by observations that are above the value given by Q3+1.5*(Q3-Q1) or below the value given by Q1-1.5*(Q3-Q1), where Q1=1st quartile or 25th percentile (P25) and Q3=3rd quartile or 75th percentile (P75). Only the values that were not classified as outliers for each variable were included.

Statistical analyses were carried out using the SAS (Statistical Analysis System) software (SAS Institute Inc., USA) for descriptive, comparative, and correlation tests.

To evaluate the seasonality of the climatic variables, the Friedman test was used and the ARIMA (autoregressive integrated moving average) was selected as the best model adjusted to the data.

Temporal analyses using the Cosinor method were carried out and tested the presence of biological rhythms in plasma lipids and lipoproteins and in cardiovascular outcomes, based on the median of the parameters analyzed throughout the eight-year period. The biological rhythm parameters determined were: MESOR (midline-estimating statistic of rhythm), amplitude (distance from MESOR to the peak of the best cosine curve fitting, approximating the data), and acrophase (timing of the peak of the best cosine curve fitting approximating the time series data in relation to a reference, 00:00 h, December 31) (14). The significance of rhythm level detection was considered with a P-value ≤0.05.

The cross-correlation function (CCF) was used to assess the correlations between variations in the climatic parameters and lipids and the rhythmicity of cardiovascular outcomes. This analysis shows how much (previous, current, or later) measurements of climatic data were associated with the other variable measured. The lag numbers indicate the number of months between the correlated variables tested and refers to the correlation between two time series relative to one another. One series may have a delay or advance in relation to the other.

## Results

### Plasma lipid and lipoprotein results

The studied population comprised 27,543 participants, totaling 228,748 laboratory tests. The mean age (means±SD) was equal to 46±16.8 years; 58% of the samples were from female individuals. The mean lipid profile (±SD, mg/dL) throughout the eight-year period was: 200.9±55.7 for cholesterol, 124.4±47.5 for LDL-C, 47.1±14.8 for HDL-C, and 146.7±119.2 for TG. The percentage frequencies of exclusive dyslipidemia in the total population were: i) 48% hypercholesterolemia (13,424 individuals, with 14,119 non-altered); ii) 43% hyperbetalipoproteinemia (11,909 individuals, with 15,634 non-altered); iii) 33% hypoalphalipoproteinemia (9,180 individuals, with 16,003 non-altered); iv) 9% hyperalphalipoproteinemia (2,360 individuals); and v) 37% hypertriglyceridemia (10,180 individuals, with 17,363 non-altered).

The inter-assay coefficients of variation (CV) for laboratory control materials used in this study were 2.4 and 1.78% for cholesterol, respectively, for non-altered and pathological controls. The analytical performances of LDL-C and HDL-C were evaluated with non-altered controls and the variation was 2.41 and 4.35%, respectively. TG presented CV equal to 2.81 and 3.02%, respectively, for non-altered and pathological controls. All these variations are much below the total error allowed by the NCEP (10).

### Variations of atherosclerotic cardiovascular disease outcomes

**Figure 1:**
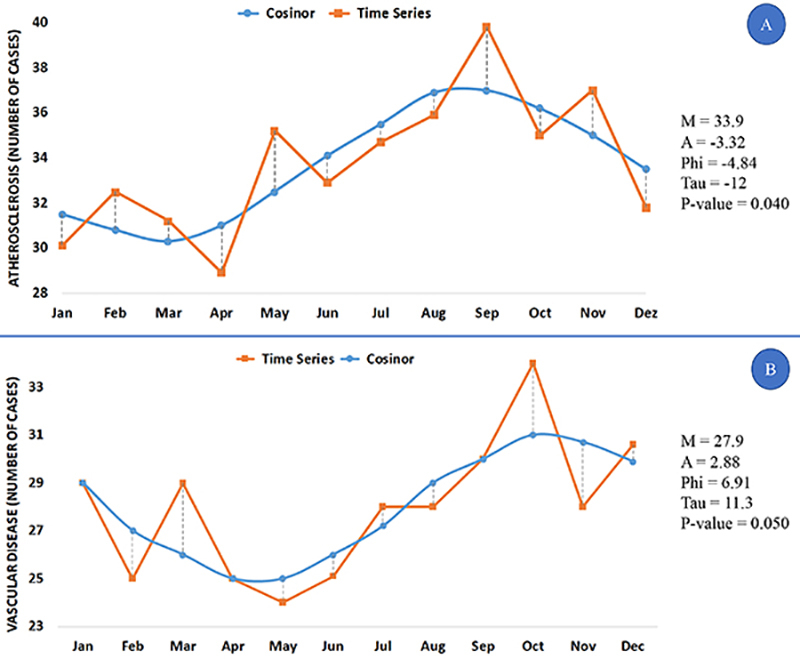
Cosinor analysis for prevalence of outcomes of atherosclerotic cardiovascular hospitalizations in Campinas, São Paulo state for 3,261 cases of atherosclerosis (**A**) and 2,685 cases of vascular disease (**B**). M: MESOR (as number of cases); A: amplitude (as number of cases); PHI: acrophase (as months of the year from January to December); TAU: period of rhythm (as months of the year); P-value: probability of biorhythms.

The Cosinor results for the number of hospitalized cases due to atherosclerotic cardiovascular disease (N=5,946, total) are shown in Figure 1 (A, atherosclerosis, N=3,261 and B, other vascular diseases, N=2,685). The number of cardiovascular hospitalizations increased from the winter to spring months. The frequency and rhythmicity of outcomes showed similar seasonal variations for atherosclerosis and other vascular diseases, with maximum frequency in winter and minimum values in summer.

### Climatic data changes

**Figure 2:**
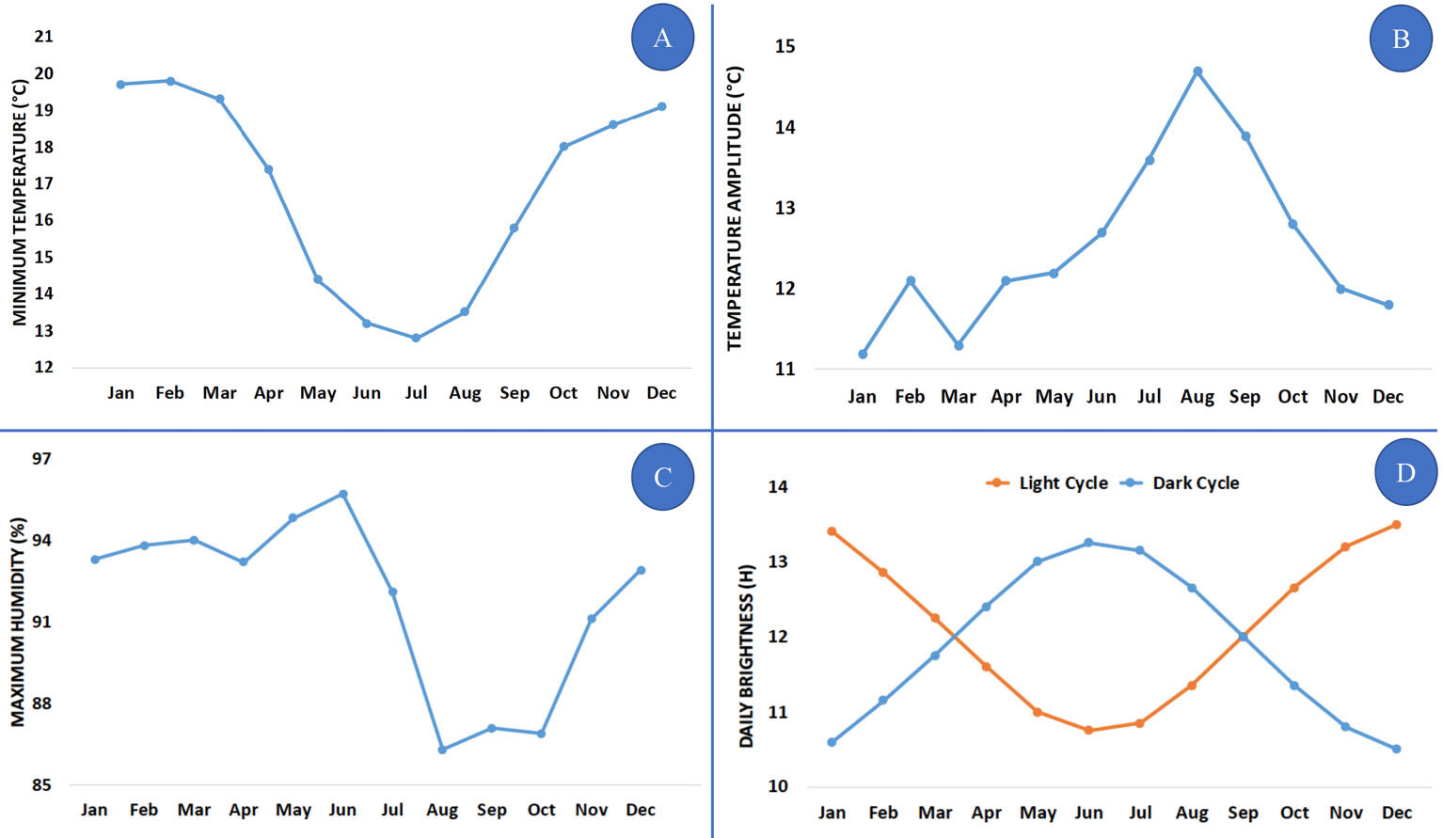
Time series analysis for minimum temperature (**A**), temperature amplitude (**B**), maximum humidity (**C**), and dark/light cycles (**D**) during the eight-year study period. The ARIMA model was used, and the following information was achieved: **A**, R^2^=0.779; spatio-temporal stationary R^2^=0.551; RMSE (root mean square error) =1.418; BIC (Bayesian information criterion)=0.805. **B**, R^2^=0.459; spatio-temporal stationary R^2^=0.236; RMSE=1.925; BIC=0.907. **C**, R^2^=0.827; spatio-temporal stationary R^2^=0.568; RMSE=1.339; BIC=0.775. **D**, (light cycle) R^2^=0.559; spatio-temporal stationary R^2^=0.331; RMSE=1.556; BIC=0.857; (dark cycle) R^2^=0.441; spatio-temporal stationary R^2^=0.356; RMSE=1.026; BIC=0.104.

Figure 2 shows the seasonal variations of the minimum temperature measurements (Figure 2A), temperature amplitude (Figure 2B), maximum humidity (Figure 2C), and dark/light cycles (Figure 2D). Also, in the southeastern region of Brazil where Campinas is located, the lowest temperature and maximum humidity, the highest temperature amplitude, and increased time of dark cycles occurred in winter (July). Opposite effects were observed in summer (January).

### Seasonal variations of lipids and lipoproteins

In addition, in the normolipidemic group (n=11,892), significant seasonal rhythmicity was observed only in plasma LDL-C (P-value=0.031; Supplementary Figure S1A) and HDL-C (P-value=0.018; Supplementary Figure S1B) in a 12-month period, with maximum values in winter and minimum values in summer. Cholesterol did not present significant rhythms. The plasma percentage concentration differences between seasons were 3.5 and 3.7%, respectively, both below the intraindividual biological variations.

**Figure 3:**
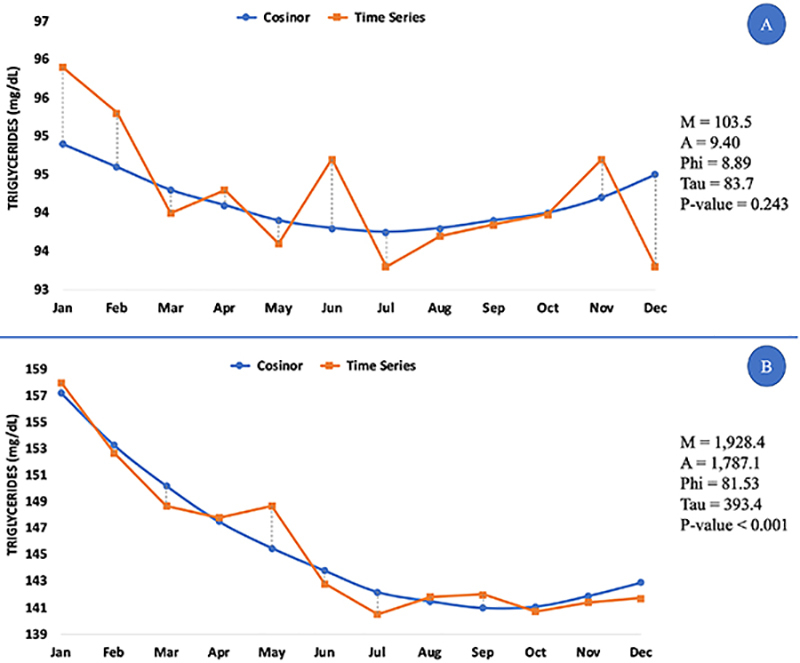
Cosinor analysis for triglycerides in normolipidemic (**A**) and dyslipidemic (**B**) individuals during the eight-year study period. Measured parameters: M: MESOR (as mg/dL); A: amplitude (as mg/dL); PHI: acrophase (as months of the year from January to December); TAU: period of rhythm (as months of the year); P-value: probability of biorhythms; n=15,651 cases of dyslipidemia.

In the dyslipidemic group (n=15,651), an increased number of seasonal rhythms was observed. Significant seasonality was seen in plasma cholesterol (P-value=0.020; Supplementary Figure S2A), LDL-C (P-value<0.001; Supplementary Figure S2B), and HDL-C (P-value=0.010; Supplementary Figure S2C) in a 12-month period, with maximum values in winter and minimum values in summer, as seen for LDL-C and HDL-C in the normolipidemic group. TG did not present significant rhythms in normolipidemic individuals (Figure 3A), however, dyslipidemic individuals presented higher values in summer and lower in winter only (Figure 3B). The plasma concentration percentage differences between winter and summer were 2.7 (C), 5.8 (LDL-C), and 8.9 (HDL-C), higher in winter, and 9.6 (TG) higher in summer; and all of which were below the intra-individual biological variations (11).

**Figure 4:**
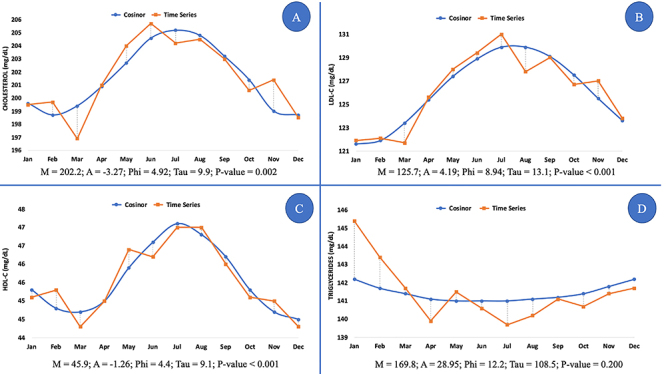
Cosinor analysis for cholesterol (**A**), LDL-C (low-density lipoprotein-cholesterol) (**B**), HDL-C (high-density lipoprotein-cholesterol) (**C**), and triglycerides (**D**) in the whole population during the eight-year study period. Measured parameters: M: MESOR (as mg/dL); A: amplitude (as mg/dL); PHI: acrophase (as months of the year from January to December); TAU: period of rhythm (as months of the year); P-value: probability of biorhythms; n=27,543 participants.

Finally, considering the whole population, the Cosinor analysis showed that the total cholesterol level and cholesterol rich-lipoprotein levels, but not TG, increased in the winter months (Figure 4A-D).

### Cross-correlation analyses of temporal series

In Table 1, we observed mostly strong correlations for CCF of minimum temperature, temperature amplitude, maximum humidity, and dark cycles variabilities with the rhythmicity of LDL-C and HDL-C; they were inverse for minimum temperature and maximum humidity and positive for temperature amplitude and dark cycles. Regarding TG, there were positive cross-correlations with minimum temperature and maximum humidity and negative cross-correlations with temperature amplitude and dark cycles.

**Table 1:** Cross-correlation functions of minimum temperature, temperature amplitude, maximum humidity, and dark cycles variability with rhythmicity of LDL-C, HDL-C, TG, and MI.

Variables	Parameters	Lag time	CCorr	Correlation strength
LDL-C	Minimum temperature with LDL-C	0	-0.884	High
LDL-C	Temperature amplitude with LDL-C	0	0.838	High
LDL-C	Maximum humidity with LDL-C	0	-0.729	High
LDL-C	Dark cycle with LDL-C	0	0.834	High
HDL-C	Minimum temperature with HDL-C	0	-0.861	High
HDL-C	Temperature amplitude with HDL-C	0	0.766	High
HDL-C	Maximum humidity with HDL-C	2	-0.650	Moderate
HDL-C	Dark cycle with HDL-C	0	0.786	High
TG	Minimum temperature with TG	0	0.512	Moderate
TG	Temperature amplitude with TG	0	-0.563	Moderate
TG	Maximum humidity with TG	0	0.715	High
TG	Dark cycle with TG	0	-0.437	Low
MI	Minimum temperature with MI	0	-0.514	Moderate
MI	Temperature amplitude with MI	0	0.550	Moderate
MI	Maximum humidity with MI	0	-0.395	Low
MI	Dark cycle with MI	0	0.457	Low

LDL-C: low-density lipoprotein-cholesterol; HDL-C: high-density lipoprotein-cholesterol; TG: triglycerides; MI: myocardial infarction; Lag time: number of months; Ccorr: cross-correlation functions.

We present some results of moderate CCF (Table 1) of minimum temperature and temperature amplitude variabilities with the rhythmicity of myocardial infarction. We observed an inverse correlation with minimum temperature and positive correlations with temperature amplitude.

The CCF identified strong correlations between the markers. Supplementary Table S1 shows a complete overview of all cross-correlations tested between climatic variables and atherosclerosis, stroke, myocardial infarction, and other vascular diseases in the total population and between climatic variables and lipids (cholesterol and TG) and lipoproteins (HDL-C and LDL-C) in normolipidemic and dyslipidemic groups.

Although the data of lipid profiles and of cardiovascular outcomes are from distinct secondary databases, we decided to evaluate the cross-correlations between lipids and hospitalizations since all individuals are from Campinas and were evaluated during the same 8-year period.

Positive cross-correlations were shown between rhythms of LDL-C and of atherosclerosis in normolipidemic individuals (Lag 2, CCorr=0.648). Conversely, dyslipidemic individuals presented more positive cross-correlations: between rhythms of LDL-C and of atherosclerosis (Lag 2, CCorr=0.727) and other vascular diseases (Lag 3, CCorr=0.819). Rhythms of cholesterol, HDL-C, and TG did not show any cross-correlations with the time series regarding the frequency of cardiovascular outcomes.

When analyzing the specific dyslipidemic group, the CCF for the rhythms of cardiovascular outcomes, lipids, and lipoproteins showed positive cross-correlations with the rhythms of LDL-C and of atherosclerosis (Lag 0, CCorr=0.820) in hypercholesterolemia, LDL-C and atherosclerosis (Lag 0, CCorr=0.691) in hyperbetalipoproteinemia, LDL-C and atherosclerosis (Lag 2, CCorr=0.662) in hyperalphalipoproteinemia, and LDL-C and myocardial infarction (Lag 0, CCorr=0.714) in hypertriglyceridemia.

## Discussion

It is important to establish the extent to which biological oscillations affect routine exams in the clinical laboratory because of their potential to alter medical decisions. This study is one of the very few that investigated the correlations of rhythms of lipids from a large population (27,543 participants) and of atherosclerotic cardiovascular hospitalizations with temporal variations of climatic variables in an eight-year period.

The lowest temperature and the lowest maximum humidity, the highest temperature amplitude, and increased dark cycles occurred in winter (July). Opposite effects were observed in summer (January), as shown in Figure 2.

Circannual variations in serum cholesterol, LDL-C, and HDL-C, with maximum values in winter and minimum ones in summer were detected. TG did not present significant changes, except in dyslipidemia with higher values during summer. Non-altered and pathological values maintained their rhythms, except for TG.

The Cosinor analysis for TG in dyslipidemic patients suggested that its rhythm occurs every 393 months and has a Mesor of 1,928 mg/dL. These values are noteworthy. They have a defined rhythm only in dyslipidemic patients. In a literature review, Ma et al. (15) indicated that TG rhythms are conflicting and have no consensus. There might be confounding factors, such as diet, drug therapy, and some pathological conditions directly influencing these seasonal rhythms of the patients evaluated in our studies.

Seasonal variation of cardiovascular outcomes with maximum values in winter and minimum values in summer were observed in parallel to the same observed oscillation of serum cholesterol, LDL-C, and HDL-C.

Similar results of circannual variations in serum lipids and lipoproteins were found by Gordon et al. (16) who studied 1,446 hypercholesterolemic men followed for seven years as a placebo group and found seasonal variations in cholesterol, LDL-C, and HDL-C with peaks in winter and a nadir in summer. TG presented irregular rhythms with maximum values in fall and minimum values in spring (16). TG rhythm is controversial in the literature and its probable origin is still unknown.

Kamezaki et al. (17) evaluated 1,331 (1,192 men and 139 women) Japanese workers, with a mean age of 43 years, in winter and summer months. Their findings indicated that the LDL-C, HDL-C, and TG parameters had their highest concentrations in the winter months and that this seasonal variation would imply an increase of 3.6% in patients diagnosed as hypercholesterolemic if their exams were performed only in the winter months (17).

To assess seasonal variation in patients with acute coronary syndromes receiving statins, Tung et al. (18) analyzed 4,162 patients and found a statistically significant difference for HDL-C during the different seasons of the year. The median HDL-C was 37 mg/dL in winter, 40 mg/dL in spring, 39 mg/dL in summer, and 36 mg/dL in fall.

Another study carried out in Brazil to assess the impact of seasonality on dyslipidemia simultaneously evaluated 227,359 individuals aged 0 to 110 years and found significant seasonal variation in total cholesterol levels, LDL-C, non-HDL-C, and HDL-C, with higher concentrations of total cholesterol, LDL-C, and HDL-C in winter and lower concentrations in summer (6).

Although it is uncertain how seasonality can influence the concentrations of lipids and lipoproteins, Zhou et al. (19) concluded that levels of cholesterol, TG, and LDL-C were higher in winter and lower in summer and that a 20°C temperature change can result in a 20% alteration in plasma lipid concentrations. These changes may be related to the incidence of cardiovascular events (20).

Corroborating our findings, Mavri et al. (20), when evaluating risk factors for coronary artery disease in 82 subjects, demonstrated significantly higher cholesterol, LDL-C, and TG in the cold months, however, with a significant reduction in HDL-C. Concomitant with lipids, other risk factors (metabolic and hemostatic) for coronary artery disease were observed in the winter months (21).

Our study also pointed out that there was an increase in the number of outcomes in the winter months, both for atherosclerosis and for vascular disease. This behavior agreed with the findings of the literature. According to Stewart et al. (21), cardiovascular event rates in winter are typically 10 to 20% higher than during the summer.

González Hernandéz et al. (22) found a seasonal rhythm in hospitalizations for acute myocardial infarction (AMI), with an increase in winter and a decrease in summer. The highest peak (acrophase) occurred in winter, with 2,183 cases (r^2^=0.91).

Yang et al. (23) found that 42% of deaths due to cardiovascular diseases and 35% of AMI deaths were caused by adverse winter temperatures, compared to 13 and 8% in summer.

It seems relevant to emphasize the seasonal rhythms of serum LDL-C and HDL-C, with higher values in winter and lower in summer, observed in this large population study, and that such annual fluctuations could impact clinical and laboratory approaches in normolipidemic and even more in dyslipidemic individuals.

In conclusion, this study in Campinas, Brazil showed for the first time that variations of temperature, humidity, and light cycle lengths are strongly associated with LDL-C and HDL-C seasonality, but moderately to lowly with rhythmicity of atherosclerotic outcomes. It also indicated unfavorable cardiovascular changes during wintertime. Further analyses are underway to examine other associations among these temporal oscillations and to explore their potential implications in the prevention of cardiovascular diseases.

## Supplementary Material

Click here to view [pdf].
